# Ethyl 1-[2-(1*H*-benzotriazol-1-yl)acet­yl]-4-hy­droxy-2,6-diphenyl-1,2,5,6-tetra­hydro­pyridine-3-carboxyl­ate

**DOI:** 10.1107/S1600536811020241

**Published:** 2011-06-04

**Authors:** G. Aridoss, S. Sundaramoorthy, D. Velmurugan, Y. T. Jeong

**Affiliations:** a Department of Image Science and Engineering, Pukyong National University, Busan 608-739, Republic of Korea; b Centre of Advanced Study in Crystallography and Biophysics, University of Madras, Guindy Campus, Chennai 600 025, India

## Abstract

In the title compound, C_28_H_26_N_4_O_4_, the tetra­hydro­pyridine ring adopts a boat conformation. The two phenyl rings form dihedral angles of 88.64 (8) and 59.28 (10)° with the best plane through the tetra­hydro­pyridine ring. The dihedral angle between the two phenyl rings is 82.55 (10)°. The benzotriazole ring system is essentially planar, with a maximum deviation of 0.009 (1) Å from the least-squares plane. The mol­ecular conformation is stabilized by an intra­molecular O—H⋯O hydrogen bond, generating an *S*(6) motif.

## Related literature

For the synthesis and medicinal properties of piperidin-4-one-based amides, see: Aridoss *et al.* (2010*a* ▶). For related structures see: Aridoss *et al.* (2010*a* ▶, 2010*b* ▶, 2011 ▶). For ring conformational analysis, see: Cremer & Pople (1975 ▶); Nardelli (1983 ▶).
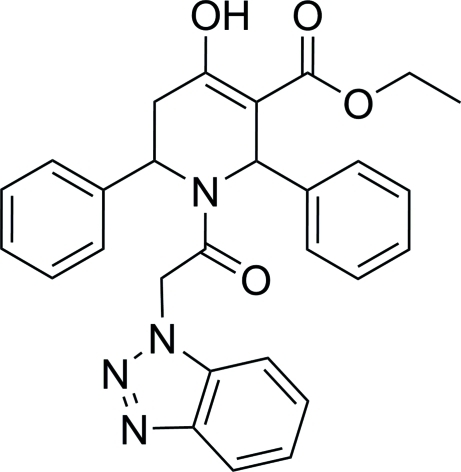


## Experimental

### 

#### Crystal data

C_28_H_26_N_4_O_4_
*M* *_r_* = 482.53
Monoclinic, 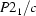

*a* = 7.9214 (2) Å
*b* = 21.9667 (6) Å
*c* = 14.5621 (4) Å
β = 95.722 (2)°
*V* = 2521.28 (12) Å^3^
*Z* = 4
Mo *K*α radiation
μ = 0.09 mm^−1^
*T* = 293 K
0.26 × 0.24 × 0.22 mm

#### Data collection

Bruker SMART APEXII area-detector diffractometer
Absorption correction: multi-scan (*SADABS*; Bruker, 2008 ▶) *T*_min_ = 0.978, *T*_max_ = 0.981
24470 measured reflections
6280 independent reflections
4096 reflections with *I* > 2σ(*I*)
*R*_int_ = 0.025

#### Refinement

*R*[*F* ^2^ > 2σ(*F* ^2^)] = 0.047
*wR*(*F* ^2^) = 0.128
*S* = 1.04
6280 reflections
326 parameters
1 restraint
H-atom parameters constrained
Δρ_max_ = 0.20 e Å^−3^
Δρ_min_ = −0.23 e Å^−3^

### 

Data collection: *APEX2* (Bruker, 2008 ▶); cell refinement: *SAINT* (Bruker, 2008 ▶); data reduction: *SAINT*; program(s) used to solve structure: *SHELXS97* (Sheldrick, 2008 ▶); program(s) used to refine structure: *SHELXL97* (Sheldrick, 2008 ▶); molecular graphics: *ORTEP-3* (Farrugia, 1997 ▶); software used to prepare material for publication: *SHELXL97* and *PLATON* (Spek, 2009 ▶).

## Supplementary Material

Crystal structure: contains datablock(s) global, I. DOI: 10.1107/S1600536811020241/bt5557sup1.cif

Structure factors: contains datablock(s) I. DOI: 10.1107/S1600536811020241/bt5557Isup2.hkl

Supplementary material file. DOI: 10.1107/S1600536811020241/bt5557Isup3.cml

Additional supplementary materials:  crystallographic information; 3D view; checkCIF report

## Figures and Tables

**Table 1:** Hydrogen-bond geometry (Å, °)

*D*—H⋯*A*	*D*—H	H⋯*A*	*D*⋯*A*	*D*—H⋯*A*
O2—H2⋯O3	0.82	1.90	2.605 (2)	143

**Fig. 1.**
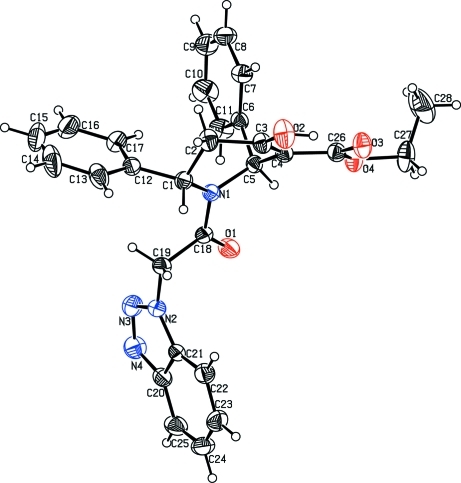
Perspective view of the molecule showing the displacement ellipsoids drawn at the 30% probability level.

**Fig. 2.**
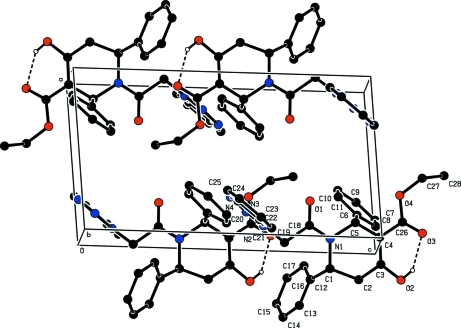
The crystal packing of the molecules viewed down the b axis. For clarity, hydrogen atoms which are not involved in hydrogen bonding are omitted.

## supplementary crystallographic information

### Comment

In continuation of our interest on the conformation and crystal studies of piperidin-4-one based amides (Aridoss *et al.* 2010*a*, 2010*b*, 2011), we are reporting herewith the crystal structure of the title compound.

The molecular structure of compound (I) is illustrated in Fig.1. The sum of the bond angles around the atom N1 [358.77 (32)°] of the tetrahydropyridine ring in the molecule is in accordance with *sp*^2^ hybridization. The two phenyl rings form dihedral angles of 88.64 (8) and 59.28 (10)° with the best plane through the tetrahydropyridine ring. The benzotriazole ring system is essentially planar with a maximum deviation of 0.009 (1)Å from the least squares plane. The ethyl acetate group shows an extended conformation [C26—O4—C27—C28 = 82.2 (2)°].

In the present structure tetrahydropyridine ring adopts a boat conformation with atoms C2 and C5 deviating by -0.316 (2) and -0.315 (1) Å, respectively from the least squares plane defined by the remaining atoms N1/C1/C3/C4 in the ring. The puckering parameters (Cremer & Pople, 1975) are Q = 0.5478 (16) Å; Θ = 90.33 (16) and Φ = 123.49 (16)°. The molecular structure is stabilized by a strong O—H···O hydrogen bond, wherein, atom O2 acts as a donor to O3, generating an *S* (6) motif.

### Experimental

The title compound was prepared from *N*-bromoacetyl-3-carboxyethyl -2,6-diphenyl-4-hydroxy-Δ^3^-tetrahydropyridine and benzotriazole according to the literature method (Aridoss *et al.*, 2010*a*). Slow evaporation of the ethanolic solution of the target compound at room temperature gave fine white crystals suitable for X-ray studies.

### Refinement

H atoms were positioned geometrically (C—H = 0.93–0.98 Å, O—H = 0.82Å) and allowed to ride on their parent atoms, with 1.5*U*_eq_(C,O) for methyl H and hydroxyl H and 1.2 *U*_eq_(C) for other H atoms. The anisotropic displacement parameters of C15 and C16 were restrained to be equal in the direction of the bond between them.

### Crystal data

**Table:**

C_28_H_26_N_4_O_4_	*F*(000) = 1016
*M**_r_* = 482.53	*D*_x_ = 1.271 Mg m^−^^3^
Monoclinic, *P*2_1_/*c*	Mo *K*α radiation, λ = 0.71073 Å
Hall symbol: -P 2ybc	Cell parameters from 1044 reflections
*a* = 7.9214 (2) Å	θ = 1.7–28.4°
*b* = 21.9667 (6) Å	µ = 0.09 mm^−^^1^
*c* = 14.5621 (4) Å	*T* = 293 K
β = 95.722 (2)°	Block, colourless
*V* = 2521.28 (12) Å^3^	0.26 × 0.24 × 0.22 mm
*Z* = 4	

### Data collection

**Table:**

Bruker SMART APEXII area-detector diffractometer	6280 independent reflections
Radiation source: fine-focus sealed tube	4096 reflections with *I* > 2σ(*I*)
graphite	*R*_int_ = 0.025
ω and φ scans	θ_max_ = 28.4°, θ_min_ = 1.7°
Absorption correction: multi-scan (*SADABS*; Bruker, 2008)	*h* = −10→9
*T*_min_ = 0.978, *T*_max_ = 0.981	*k* = −29→21
24470 measured reflections	*l* = −18→19

### Refinement

**Table:**

Refinement on *F*^2^	Primary atom site location: structure-invariant direct methods
Least-squares matrix: full	Secondary atom site location: difference Fourier map
*R*[*F*^2^ > 2σ(*F*^2^)] = 0.047	Hydrogen site location: inferred from neighbouring sites
*wR*(*F*^2^) = 0.128	H-atom parameters constrained
*S* = 1.04	*w* = 1/[σ^2^(*F*_o_^2^) + (0.0508*P*)^2^ + 0.4558*P*] where *P* = (*F*_o_^2^ + 2*F*_c_^2^)/3
6280 reflections	(Δ/σ)_max_ = 0.001
326 parameters	Δρ_max_ = 0.20 e Å^−^^3^
1 restraint	Δρ_min_ = −0.23 e Å^−^^3^

### Special details

**Table:**

Geometry. All e.s.d.'s (except the e.s.d. in the dihedral angle between two l.s. planes) are estimated using the full covariance matrix. The cell e.s.d.'s are taken into account individually in the estimation of e.s.d.'s in distances, angles and torsion angles; correlations between e.s.d.'s in cell parameters are only used when they are defined by crystal symmetry. An approximate (isotropic) treatment of cell e.s.d.'s is used for estimating e.s.d.'s involving l.s. planes.
Refinement. Refinement of *F*^2^ against ALL reflections. The weighted *R*-factor *wR* and goodness of fit *S* are based on *F*^2^, conventional *R*-factors *R* are based on *F*, with *F* set to zero for negative *F*^2^. The threshold expression of *F*^2^ > σ(*F*^2^) is used only for calculating *R*-factors(gt) *etc*. and is not relevant to the choice of reflections for refinement. *R*-factors based on *F*^2^ are statistically about twice as large as those based on *F*, and *R*- factors based on ALL data will be even larger.

### Fractional atomic coordinates and isotropic or equivalent isotropic displacement parameters (Å^2^)

**Table:**

	*x*	*y*	*z*	*U*_iso_*/*U*_eq_	
C14	−0.4394 (4)	0.29971 (18)	0.66570 (15)	0.1189 (12)	
H14	−0.5497	0.3046	0.6384	0.143*	
C28	0.4568 (4)	0.41609 (15)	1.23886 (19)	0.1343 (11)	
H28A	0.3484	0.4043	1.2572	0.201*	
H28B	0.5241	0.4337	1.2906	0.201*	
H28C	0.5137	0.3809	1.2178	0.201*	
C1	−0.13796 (17)	0.38583 (7)	0.83335 (10)	0.0444 (3)	
H1	−0.1908	0.4243	0.8122	0.053*	
C2	−0.19984 (19)	0.37148 (9)	0.92782 (10)	0.0548 (4)	
H2A	−0.3192	0.3818	0.9260	0.066*	
H2B	−0.1888	0.3281	0.9393	0.066*	
C3	−0.10443 (19)	0.40503 (7)	1.00537 (10)	0.0494 (4)	
C4	0.06433 (17)	0.41019 (7)	1.00816 (9)	0.0428 (3)	
C5	0.15248 (17)	0.38475 (6)	0.92983 (9)	0.0398 (3)	
H5	0.2567	0.4085	0.9274	0.048*	
C6	0.20532 (17)	0.31828 (7)	0.93869 (9)	0.0423 (3)	
C7	0.1435 (2)	0.27823 (8)	1.00015 (11)	0.0535 (4)	
H7	0.0707	0.2924	1.0417	0.064*	
C8	0.1882 (2)	0.21739 (8)	1.00086 (13)	0.0646 (5)	
H8	0.1433	0.1909	1.0419	0.078*	
C9	0.2973 (2)	0.19597 (9)	0.94192 (13)	0.0696 (5)	
H9	0.3261	0.1549	0.9421	0.084*	
C10	0.3643 (3)	0.23537 (9)	0.88223 (14)	0.0745 (5)	
H10	0.4410	0.2212	0.8428	0.089*	
C11	0.3185 (2)	0.29595 (8)	0.88043 (11)	0.0605 (4)	
H11	0.3644	0.3222	0.8394	0.073*	
C12	−0.2044 (2)	0.33717 (8)	0.76537 (10)	0.0530 (4)	
C13	−0.3676 (2)	0.34412 (11)	0.72253 (11)	0.0769 (6)	
H13	−0.4286	0.3793	0.7325	0.092*	
C15	−0.3517 (5)	0.24853 (17)	0.64860 (16)	0.1274 (14)	
H15	−0.4011	0.2189	0.6090	0.153*	
C16	−0.1890 (4)	0.24064 (11)	0.69020 (15)	0.1051 (9)	
H16	−0.1288	0.2055	0.6789	0.126*	
C17	−0.1147 (3)	0.28529 (9)	0.74913 (12)	0.0728 (5)	
H17	−0.0051	0.2800	0.7773	0.087*	
C18	0.12276 (18)	0.42430 (7)	0.77372 (10)	0.0428 (3)	
C19	0.01021 (19)	0.43927 (7)	0.68500 (10)	0.0492 (4)	
H19A	−0.0417	0.4022	0.6594	0.059*	
H19B	−0.0797	0.4667	0.6991	0.059*	
C20	0.25001 (19)	0.52618 (8)	0.53605 (10)	0.0494 (4)	
C21	0.13955 (17)	0.52698 (7)	0.60430 (9)	0.0418 (3)	
C22	0.0838 (2)	0.58034 (8)	0.64184 (11)	0.0565 (4)	
H22	0.0102	0.5804	0.6877	0.068*	
C23	0.1446 (3)	0.63310 (8)	0.60675 (14)	0.0695 (5)	
H23	0.1105	0.6702	0.6295	0.083*	
C24	0.2553 (3)	0.63297 (10)	0.53843 (14)	0.0735 (6)	
H24	0.2928	0.6700	0.5170	0.088*	
C25	0.3104 (2)	0.58077 (9)	0.50207 (13)	0.0666 (5)	
H25	0.3850	0.5812	0.4566	0.080*	
C26	0.15896 (19)	0.43862 (7)	1.08725 (10)	0.0461 (3)	
C27	0.4324 (2)	0.46121 (10)	1.16347 (14)	0.0777 (6)	
H27A	0.3804	0.4976	1.1858	0.093*	
H27B	0.5418	0.4724	1.1441	0.093*	
N1	0.04728 (13)	0.39513 (5)	0.84073 (7)	0.0389 (3)	
N2	0.10739 (15)	0.46717 (5)	0.61823 (8)	0.0451 (3)	
N3	0.19402 (19)	0.43209 (6)	0.56320 (9)	0.0594 (4)	
N4	0.27962 (19)	0.46712 (7)	0.51253 (10)	0.0642 (4)	
O1	0.27280 (13)	0.43786 (6)	0.78083 (7)	0.0613 (3)	
O2	−0.19961 (14)	0.42665 (6)	1.06894 (8)	0.0720 (4)	
H2	−0.1387	0.4440	1.1097	0.108*	
O3	0.09123 (14)	0.46188 (6)	1.15100 (8)	0.0624 (3)	
O4	0.32511 (13)	0.43677 (5)	1.08484 (8)	0.0593 (3)	

### Atomic displacement parameters (Å^2^)

**Table:**

	*U*^11^	*U*^22^	*U*^33^	*U*^12^	*U*^13^	*U*^23^
C14	0.0988 (19)	0.204 (3)	0.0527 (12)	−0.091 (2)	0.0002 (12)	−0.0100 (17)
C28	0.116 (2)	0.176 (3)	0.0982 (19)	0.026 (2)	−0.0519 (16)	0.007 (2)
C1	0.0371 (7)	0.0475 (9)	0.0476 (8)	−0.0058 (6)	0.0002 (6)	0.0031 (6)
C2	0.0403 (8)	0.0726 (11)	0.0522 (9)	−0.0109 (8)	0.0084 (7)	−0.0044 (8)
C3	0.0456 (8)	0.0587 (10)	0.0445 (8)	−0.0008 (7)	0.0074 (6)	−0.0034 (7)
C4	0.0420 (8)	0.0444 (8)	0.0419 (7)	−0.0002 (6)	0.0028 (6)	0.0002 (6)
C5	0.0356 (7)	0.0448 (8)	0.0385 (7)	−0.0049 (6)	0.0013 (5)	0.0004 (6)
C6	0.0389 (7)	0.0469 (9)	0.0401 (7)	0.0009 (6)	−0.0007 (6)	0.0028 (6)
C7	0.0539 (9)	0.0536 (10)	0.0536 (9)	−0.0010 (8)	0.0082 (7)	0.0083 (7)
C8	0.0665 (11)	0.0542 (11)	0.0715 (11)	−0.0046 (9)	−0.0020 (9)	0.0170 (9)
C9	0.0764 (12)	0.0499 (11)	0.0795 (12)	0.0128 (9)	−0.0079 (10)	0.0050 (9)
C10	0.0820 (13)	0.0678 (13)	0.0755 (12)	0.0280 (11)	0.0164 (10)	0.0026 (10)
C11	0.0633 (10)	0.0610 (11)	0.0592 (9)	0.0139 (9)	0.0171 (8)	0.0111 (8)
C12	0.0559 (9)	0.0623 (11)	0.0405 (7)	−0.0247 (8)	0.0032 (7)	0.0053 (7)
C13	0.0573 (10)	0.1251 (18)	0.0471 (9)	−0.0358 (11)	−0.0001 (8)	0.0049 (10)
C15	0.179 (3)	0.154 (3)	0.0506 (12)	−0.122 (3)	0.0183 (16)	−0.0240 (15)
C16	0.178 (3)	0.0749 (15)	0.0656 (13)	−0.0515 (17)	0.0259 (16)	−0.0143 (11)
C17	0.1006 (15)	0.0570 (12)	0.0597 (10)	−0.0217 (11)	0.0022 (10)	−0.0040 (9)
C18	0.0433 (8)	0.0402 (8)	0.0446 (7)	−0.0058 (6)	0.0024 (6)	0.0036 (6)
C19	0.0505 (8)	0.0508 (9)	0.0455 (8)	−0.0100 (7)	0.0009 (6)	0.0094 (7)
C20	0.0462 (8)	0.0579 (10)	0.0433 (7)	−0.0033 (7)	0.0015 (6)	0.0067 (7)
C21	0.0437 (8)	0.0430 (9)	0.0372 (7)	−0.0018 (6)	−0.0032 (6)	0.0053 (6)
C22	0.0633 (10)	0.0515 (10)	0.0540 (9)	0.0050 (8)	0.0019 (8)	0.0000 (8)
C23	0.0813 (13)	0.0445 (10)	0.0782 (12)	0.0028 (9)	−0.0145 (10)	0.0011 (9)
C24	0.0757 (13)	0.0596 (12)	0.0814 (13)	−0.0179 (10)	−0.0118 (11)	0.0254 (10)
C25	0.0570 (10)	0.0812 (14)	0.0611 (10)	−0.0143 (10)	0.0030 (8)	0.0217 (10)
C26	0.0475 (8)	0.0444 (9)	0.0456 (8)	0.0028 (7)	0.0007 (6)	−0.0006 (6)
C27	0.0506 (10)	0.0928 (15)	0.0853 (13)	0.0035 (10)	−0.0154 (9)	−0.0374 (12)
N1	0.0364 (6)	0.0406 (7)	0.0394 (6)	−0.0038 (5)	0.0015 (5)	0.0034 (5)
N2	0.0535 (7)	0.0418 (7)	0.0398 (6)	−0.0020 (6)	0.0036 (5)	0.0028 (5)
N3	0.0711 (9)	0.0511 (8)	0.0566 (8)	0.0027 (7)	0.0089 (7)	−0.0066 (7)
N4	0.0684 (9)	0.0687 (10)	0.0580 (8)	0.0024 (8)	0.0187 (7)	−0.0040 (7)
O1	0.0471 (6)	0.0798 (8)	0.0560 (6)	−0.0188 (6)	0.0006 (5)	0.0189 (6)
O2	0.0507 (7)	0.1067 (10)	0.0603 (7)	0.0038 (7)	0.0141 (5)	−0.0228 (7)
O3	0.0593 (7)	0.0738 (8)	0.0534 (6)	0.0082 (6)	0.0018 (5)	−0.0188 (6)
O4	0.0438 (6)	0.0738 (8)	0.0586 (6)	−0.0018 (6)	−0.0035 (5)	−0.0157 (6)

### Geometric parameters (Å, °)

**Table:**

C14—C15	1.357 (4)	C12—C13	1.386 (2)
C14—C13	1.366 (3)	C13—H13	0.9300
C14—H14	0.9300	C15—C16	1.379 (4)
C28—C27	1.477 (3)	C15—H15	0.9300
C28—H28A	0.9600	C16—C17	1.394 (3)
C28—H28B	0.9600	C16—H16	0.9300
C28—H28C	0.9600	C17—H17	0.9300
C1—N1	1.4748 (17)	C18—O1	1.2196 (16)
C1—C12	1.515 (2)	C18—N1	1.3551 (17)
C1—C2	1.538 (2)	C18—C19	1.531 (2)
C1—H1	0.9800	C19—N2	1.4362 (18)
C2—C3	1.490 (2)	C19—H19A	0.9700
C2—H2A	0.9700	C19—H19B	0.9700
C2—H2B	0.9700	C20—N4	1.368 (2)
C3—O2	1.3379 (17)	C20—C21	1.388 (2)
C3—C4	1.338 (2)	C20—C25	1.400 (2)
C4—C26	1.451 (2)	C21—N2	1.3574 (18)
C4—C5	1.5031 (19)	C21—C22	1.384 (2)
C5—N1	1.4882 (16)	C22—C23	1.373 (2)
C5—C6	1.521 (2)	C22—H22	0.9300
C5—H5	0.9800	C23—C24	1.390 (3)
C6—C7	1.379 (2)	C23—H23	0.9300
C6—C11	1.384 (2)	C24—C25	1.353 (3)
C7—C8	1.382 (2)	C24—H24	0.9300
C7—H7	0.9300	C25—H25	0.9300
C8—C9	1.361 (3)	C26—O3	1.2289 (17)
C8—H8	0.9300	C26—O4	1.3207 (18)
C9—C10	1.371 (3)	C27—O4	1.4586 (19)
C9—H9	0.9300	C27—H27A	0.9700
C10—C11	1.379 (2)	C27—H27B	0.9700
C10—H10	0.9300	N2—N3	1.3478 (17)
C11—H11	0.9300	N3—N4	1.3024 (19)
C12—C17	1.376 (3)	O2—H2	0.8200
			
C15—C14—C13	120.9 (3)	C12—C13—H13	119.8
C15—C14—H14	119.6	C14—C15—C16	119.8 (2)
C13—C14—H14	119.6	C14—C15—H15	120.1
C27—C28—H28A	109.5	C16—C15—H15	120.1
C27—C28—H28B	109.5	C15—C16—C17	120.0 (3)
H28A—C28—H28B	109.5	C15—C16—H16	120.0
C27—C28—H28C	109.5	C17—C16—H16	120.0
H28A—C28—H28C	109.5	C12—C17—C16	119.7 (2)
H28B—C28—H28C	109.5	C12—C17—H17	120.2
N1—C1—C12	115.08 (12)	C16—C17—H17	120.2
N1—C1—C2	111.51 (11)	O1—C18—N1	123.41 (13)
C12—C1—C2	108.59 (12)	O1—C18—C19	119.99 (12)
N1—C1—H1	107.1	N1—C18—C19	116.60 (12)
C12—C1—H1	107.1	N2—C19—C18	110.90 (12)
C2—C1—H1	107.1	N2—C19—H19A	109.5
C3—C2—C1	113.18 (12)	C18—C19—H19A	109.5
C3—C2—H2A	108.9	N2—C19—H19B	109.5
C1—C2—H2A	108.9	C18—C19—H19B	109.5
C3—C2—H2B	108.9	H19A—C19—H19B	108.0
C1—C2—H2B	108.9	N4—C20—C21	109.10 (13)
H2A—C2—H2B	107.8	N4—C20—C25	130.60 (16)
O2—C3—C4	125.39 (14)	C21—C20—C25	120.29 (16)
O2—C3—C2	114.95 (13)	N2—C21—C22	133.52 (14)
C4—C3—C2	119.64 (13)	N2—C21—C20	103.65 (13)
C3—C4—C26	119.73 (13)	C22—C21—C20	122.82 (14)
C3—C4—C5	118.93 (13)	C23—C22—C21	115.50 (16)
C26—C4—C5	121.32 (12)	C23—C22—H22	122.3
N1—C5—C4	110.03 (11)	C21—C22—H22	122.3
N1—C5—C6	110.27 (11)	C22—C23—C24	122.29 (18)
C4—C5—C6	115.93 (11)	C22—C23—H23	118.9
N1—C5—H5	106.7	C24—C23—H23	118.9
C4—C5—H5	106.7	C25—C24—C23	122.18 (17)
C6—C5—H5	106.7	C25—C24—H24	118.9
C7—C6—C11	117.74 (14)	C23—C24—H24	118.9
C7—C6—C5	123.70 (13)	C24—C25—C20	116.91 (17)
C11—C6—C5	118.55 (13)	C24—C25—H25	121.5
C6—C7—C8	120.94 (16)	C20—C25—H25	121.5
C6—C7—H7	119.5	O3—C26—O4	122.96 (14)
C8—C7—H7	119.5	O3—C26—C4	123.27 (14)
C9—C8—C7	120.52 (17)	O4—C26—C4	113.76 (13)
C9—C8—H8	119.7	O4—C27—C28	111.05 (19)
C7—C8—H8	119.7	O4—C27—H27A	109.4
C8—C9—C10	119.48 (17)	C28—C27—H27A	109.4
C8—C9—H9	120.3	O4—C27—H27B	109.4
C10—C9—H9	120.3	C28—C27—H27B	109.4
C9—C10—C11	120.23 (18)	H27A—C27—H27B	108.0
C9—C10—H10	119.9	C18—N1—C1	121.35 (11)
C11—C10—H10	119.9	C18—N1—C5	116.83 (11)
C10—C11—C6	121.05 (16)	C1—N1—C5	120.59 (10)
C10—C11—H11	119.5	N3—N2—C21	110.50 (12)
C6—C11—H11	119.5	N3—N2—C19	119.84 (13)
C17—C12—C13	119.23 (17)	C21—N2—C19	129.45 (13)
C17—C12—C1	123.00 (15)	N4—N3—N2	108.89 (13)
C13—C12—C1	117.64 (17)	N3—N4—C20	107.85 (13)
C14—C13—C12	120.5 (3)	C3—O2—H2	109.5
C14—C13—H13	119.8	C26—O4—C27	118.16 (13)
			
N1—C1—C2—C3	35.76 (19)	N4—C20—C21—C22	−179.28 (14)
C12—C1—C2—C3	163.58 (14)	C25—C20—C21—C22	0.1 (2)
C1—C2—C3—O2	139.28 (15)	N2—C21—C22—C23	−178.43 (15)
C1—C2—C3—C4	−42.3 (2)	C20—C21—C22—C23	0.3 (2)
O2—C3—C4—C26	3.2 (2)	C21—C22—C23—C24	−0.3 (3)
C2—C3—C4—C26	−175.13 (14)	C22—C23—C24—C25	0.0 (3)
O2—C3—C4—C5	−178.42 (14)	C23—C24—C25—C20	0.3 (3)
C2—C3—C4—C5	3.3 (2)	N4—C20—C25—C24	178.81 (17)
C3—C4—C5—N1	37.76 (18)	C21—C20—C25—C24	−0.4 (2)
C26—C4—C5—N1	−143.84 (13)	C3—C4—C26—O3	−4.3 (2)
C3—C4—C5—C6	−88.21 (17)	C5—C4—C26—O3	177.29 (14)
C26—C4—C5—C6	90.18 (16)	C3—C4—C26—O4	175.16 (14)
N1—C5—C6—C7	−110.01 (15)	C5—C4—C26—O4	−3.2 (2)
C4—C5—C6—C7	15.84 (19)	O1—C18—N1—C1	173.04 (14)
N1—C5—C6—C11	68.27 (16)	C19—C18—N1—C1	−8.0 (2)
C4—C5—C6—C11	−165.88 (13)	O1—C18—N1—C5	5.6 (2)
C11—C6—C7—C8	−2.5 (2)	C19—C18—N1—C5	−175.37 (12)
C5—C6—C7—C8	175.82 (14)	C12—C1—N1—C18	74.41 (17)
C6—C7—C8—C9	1.3 (3)	C2—C1—N1—C18	−161.35 (13)
C7—C8—C9—C10	0.7 (3)	C12—C1—N1—C5	−118.65 (14)
C8—C9—C10—C11	−1.5 (3)	C2—C1—N1—C5	5.59 (18)
C9—C10—C11—C6	0.3 (3)	C4—C5—N1—C18	125.57 (13)
C7—C6—C11—C10	1.6 (2)	C6—C5—N1—C18	−105.31 (14)
C5—C6—C11—C10	−176.74 (16)	C4—C5—N1—C1	−41.94 (16)
N1—C1—C12—C17	33.9 (2)	C6—C5—N1—C1	87.18 (14)
C2—C1—C12—C17	−91.91 (17)	C22—C21—N2—N3	179.68 (16)
N1—C1—C12—C13	−150.30 (13)	C20—C21—N2—N3	0.79 (15)
C2—C1—C12—C13	83.93 (17)	C22—C21—N2—C19	−5.6 (3)
C15—C14—C13—C12	−1.3 (3)	C20—C21—N2—C19	175.49 (13)
C17—C12—C13—C14	0.8 (3)	C18—C19—N2—N3	83.85 (16)
C1—C12—C13—C14	−175.23 (16)	C18—C19—N2—C21	−90.42 (18)
C13—C14—C15—C16	1.1 (4)	C21—N2—N3—N4	−1.09 (17)
C14—C15—C16—C17	−0.4 (4)	C19—N2—N3—N4	−176.37 (13)
C13—C12—C17—C16	−0.1 (3)	N2—N3—N4—C20	0.89 (17)
C1—C12—C17—C16	175.68 (15)	C21—C20—N4—N3	−0.40 (18)
C15—C16—C17—C12	−0.1 (3)	C25—C20—N4—N3	−179.65 (16)
O1—C18—C19—N2	2.0 (2)	O3—C26—O4—C27	2.7 (2)
N1—C18—C19—N2	−177.02 (12)	C4—C26—O4—C27	−176.79 (15)
N4—C20—C21—N2	−0.24 (16)	C28—C27—O4—C26	82.2 (2)
C25—C20—C21—N2	179.10 (13)		

### Hydrogen-bond geometry (Å, °)

**Table:**

*D*—H···*A*	*D*—H	H···*A*	*D*···*A*	*D*—H···*A*
O2—H2···O3	0.82	1.90	2.605 (2)	143
